# Obesity and lipid metabolism disorders determine the risk for development of long COVID syndrome: a cross-sectional study from 50,402 COVID-19 patients

**DOI:** 10.1007/s15010-022-01784-0

**Published:** 2022-03-30

**Authors:** Sven H. Loosen, Björn-Erik Ole Jensen, Christian Tanislav, Tom Luedde, Christoph Roderburg, Karel Kostev

**Affiliations:** 1 Clinic for Gastroenterology, Hepatology and Infectious Diseases, University Hospital Düsseldorf, Medical Faculty of Heinrich Heine University Düsseldorf, Moorenstraße 5, 40225 Düsseldorf, Germany; 2 Department of Geriatrics and Neurology, Diakonie Hospital Jung Stilling, Siegen, Germany; 3 Epidemiology, QVIA, Frankfurt, Germany

**Keywords:** LCS, Long COVID, Post-COVID syndrome, SARS-CoV-2, BMI, Diabetes

## Abstract

**Purpose:**

Metabolic disorders have been identified as major risk factors for severe acute courses of COVID-19. With decreasing numbers of infections in many countries, the long COVID syndrome (LCS) represents the next major challenge in pandemic management, warranting the precise definition of risk factors for LCS development.

**Methods:**

We identified 50,402 COVID-19 patients in the Disease Analyzer database (IQVIA) featuring data from 1056 general practices in Germany. Multivariate logistic regression analysis was used to identify risk factors for the development of LCS.

**Results:**

Of the 50,402 COVID-19 patients included into this analysis, 1,708 (3.4%) were diagnosed with LCS. In a multivariate regression analysis, we identified lipid metabolism disorders (OR 1.46, 95% CI 1.28–1.65, *p *< 0.001) and obesity (OR 1.25, 95% CI 1.08–1.44, *p *= 0.003) as strong risk factors for the development of LCS. Besides these metabolic factors, patients’ age between 46 and 60 years (compared to age ≤ 30, (OR 1.81 95% CI 1.54–2.13, *p *< 0.001), female sex (OR 1.33, 95% CI 1.20–1.47, *p *< 0.001) as well as pre-existing asthma (OR 1.67, 95% CI 1.39–2.00, *p *< 0.001) and depression (OR 1.27, 95% CI 1.09–1.47, *p *= < 0.002) in women, and cancer (OR 1.4, 95% CI 1.09–1.95, *p *= < 0.012) in men were associated with an increased likelihood of developing LCS.

**Conclusion:**

Lipid metabolism disorders and obesity represent age-independent risk factors for the development of LCS, suggesting that metabolic alterations determine the risk for unfavorable disease courses along all phases of COVID-19.

## Introduction

The SARS-CoV-2 pandemic represents an unprecedented global challenge. By November 2021, over 247 million confirmed cases of SARS-CoV-2 have been reported and more than 5 million patients have died in association with the coronavirus disease 2019 (COVID-19) [1]. Even with infection rates and numbers of patients hospitalized for COVID-19 decreasing in some countries, the long-term consequences of SARS-CoV-2 infections, often referred to as long COVID syndrome (LCS), represent a growing medical and socioeconomic problem, worldwide [2].

The LCS can affect a wide range of organ systems such as the respiratory system or the nervous system [3]. Commonly observed symptoms include shortness of breath, fatigue, anosmia, muscle weakness or cognitive impairment [3]. However, a broad variety of at least partly unspecific symptoms have been described in the context of LCS and LCS has only been poorly defined to date and systematic data on incidence rates are largely missing [4, 5]. Current data from the United Kingdome and the United States of America indicate that the incidence of the LCS range between 7 and 13.3%, depending on the definition of LCS as well as the length of the follow-up period after initial diagnosis of COVID-19 [4, 5]. The WHO has recently published a clinical case definition of post-COVID-19 syndrome, which also includes a review of several other definitions of LCS/post-COVID syndrome [6]

Risk factors for LCS are widely unclear. In particular, it is only poorly understood if the risks for incidence and severity of LCS correlate with disease severity of acute SARS-CoV-2 infection, warranting a clear definition of risk factors for the development of LCS [2]. In addition, most existing data on LCS are primarily focusing on patients hospitalized for COVID-19, while less severe courses that are treated by general practitioners only are less frequently considered. In the present study, we, therefore, used the Disease Analyzer database (IQVIA), which features diagnoses and basic medical as well as demographic data of outpatients treated in general practices in Germany, to study the prevalence of LCS in Germany and to identify clinical factors associated with its development.

## Materials and methods

### Study design and database

This retrospective observational study was based on cross-sectional medical record data from the Disease Analyzer database (IQVIA), which compiles diagnoses as well as general medical and demographic data that are anonymously obtained from computer systems of general practitioners and specialists in Germany [7]. The sampling method for the Disease Analyzer database is based on summary statistics from all medical doctors in Germany that are published yearly by the German Medical Association and is defined according to the specialist group, the German federal state, the community size category, and the physicians’ age. The database covers ~ 3% of all outpatient practices in Germany. The sampling methods used to select physicians’ practices have been shown to be appropriate for obtaining a population-representative database of primary and specialized care in Germany [7]. Diagnoses [(according to the International Classification of Diseases, 10th revision (ICD-10)], prescriptions (according to the Anatomical Therapeutic Chemical (ATC) Classification system), and the quality of reported data are constantly monitored by IQVIA.

### Study population and outcomes

The analysis included 50,402 patients with a confirmed diagnosis of COVID-19 (ICD-10: U07.1) between March 1, 2020 and March 31, 2021 (index date) from one of 1056 GP practices that routinely send data to the Disease Analyzer database. The study’s primary outcome was the proportion of patients with a documentation of long COVID syndrome (LCS) or a diagnosis suggestive for LCS. Since there was no specific ICD-10 code for LCS during this period of time, LCS was identified based on the original diagnosis text of the physicians (“long COVID syndrome”, “post COVID syndrome, “post COVID complications”). The following ICD-10 diagnoses were additionally used as surrogates for LCS: chronic fatigue (ICD-10: G93.3), abnormalities of breathing (ICD-10: R06), disturbances of smell and taste (ICD-10: R43), malaise and fatigue (ICD-10: R53, disturbances in attention (ICD-10: R41.8). Patients with a diagnosis of one or more of these diagnoses documented within the time period between 90 and 183 days after the diagnosis of COVID-19 were enrolled. Patients with a diagnosis of one or more of these diagnoses within 12 months prior to diagnosis of COVID-19 were excluded.

### Statistical analyses

The proportion of patients with LCS was analyzed for the total study population as well as for men, women and four age groups (≤ 30, 31–45, 46–60 and > 60 years). The association between predefined variables and the incidence of LCS was investigated in a multivariable logistic regression model. This model included age, sex, and the following diagnoses documented within 12 months prior to the index date: arterial hypertension (ICD-10: I10), lipid metabolism disorders (ICD-10: E78), obesity (ICD-10: E66), cancer (ICD-10: C00–C99), type 1 diabetes mellitus (ICD-10: E10), type 2 diabetes mellitus (ICD-10: E11, E14), depression (ICD-10: F32, F33), asthma (ICD-10: J45), and chronic obstructive bronchitis or lung disease (ICD-10: J42–J44). In a subgroup of patients with available body mass index (BMI) values documented within 6 months prior to the index date (*n* = 7732), the association between BMI and LCS was analyzed in a second multivariable logistic regression model. Results from the logistic regression analyses are shown as odds ratios (ORs) and 95% confidence intervals (CI). A *p* value lower than 0.05 was considered statistically significant. All analyses were performed using SAS 9.4. (Cary, NC: SAS Institute Inc).

## Results

### Characteristics of study cohort

To identify risk factors for the development of long COVID syndrome (LCS), we performed a retrospective observational study based on cross-sectional medical record data from the Disease Analyzer database (IQVIA), which compiles diagnoses as well as general medical and demographic data obtained anonymously from computer systems of general practitioners in Germany [7]. Of the 50,402 patients with a confirmed SARS-CoV-2 infection (ICD-10: U07.1), 1708 (3.4%) were diagnosed with LCS or one of the related diagnoses (ICD-10: G93.3, R06, R43, R53; Table 1). The average time between the diagnosis of COVID-19 and the diagnosis of LCS was 82 days (SD 28 days). Each patient had a least one diagnosis of LCS or the related diagnoses > 90 days after the initial diagnosis of COVID-19. The mean age of all COVID-19 patients was 48.8 years (SD: 19.3 years. 27,512 (54.5%) of patients were female. Arterial hypertension (*n* = 12,898, 25.6%) was the most prevalent comorbidity, followed by lipid metabolism disorders (*n* = 8580, 17.0%), depression (*n* = 8529, 16.9%), diabetes mellitus type 2 (*n* = 5060, 10.0%), obesity (*n* = 4995, 9.90%), and chronic bronchitis or chronic obstructive pulmonary disease (*n* = 4399, 8.7%).

**Table 1:** Baseline characteristics of the study sample

Variable	Category	Number of patients	Proportion (%)
*n*		50,402	
Age in years	Mean (standard deviation)	48.8 (19.3)	
	≤ 30	10,443	20.7
	31–45	12,963	25.7
	46–60	14,424	28.6
	> 60	12,572	25.0
Sex	Female	27,512	54.5
	Male	22,890	45.5
Comorbidities documented within 12 months prior to the index date	Hypertension	12,898	25.6
	Lipid metabolism disorder	8,580	17.0
	Diabetes mellitus type 1	364	0.7
	Diabetes mellitus type 2	5060	10.0
	Obesity	4995	9.9
	Ischemic heart diseases	3803	7.6
	Asthma	4073	8.1
	Chronic bronchitis/COPD	4399	8.7
	Cancer	2605	5.2
	Depression	8529	16.9
Month of the first COVID-19 diagnosis	March–September 2020	7340	14.6
	October 2020	4351	8.6
	November 2020	9437	18.7
	December 2020	11,347	22.5
	January 2021	8252	16.4
	February 2021	4032	8.0
	March 2021	5643	11.2

Data represent percentages unless otherwise stated

## Clinical factors associated with the development of long COVID syndrome

To identify independent risk factors for LCS, we performed multivariate logistic regression analyses (Table 2). These analyses revealed that lipid metabolism disorders (OR 1.46, 95% CI 1.28–1.65, *p *< 0.001) and obesity (OR 1.25 95% CI 1.08–1.44, *p *= 0.003) displayed a strong association with the development of LCS. Notably, the age group between 46 and 60 years (OR 1.81, 95% CI 1.54–2.13, *p *< 0.001) was associated with a 1.8-fold higher risk of LCS compared to patients ≤ 30 years. Moreover, the risk for LCS rose gradually with increasing BMI and was highest among patients with a BMI ≥ 35 kg/m^2^; however, this association was not significant due to the small sample sizes of documented BMI values. Besides these metabolic factors, we identified that female sex (OR 1.33, 95% CI 1.20–1.47, *p *< 0.001) was significantly associated with the likelihood of being diagnosed with LCS. In addition, pre-existing asthma (OR 1.49, 95% CI 1.28–1.73, *p *< 0.001), hypertension (OR 1.31, 95% CI 1.15–1.48, *p *< 0.001), and depression (OR 1.21, 95% CI 1.07–1.37, *p *= 0.002) turned out as risk factors for the development of LCS. In contrast, pre-existing diabetes mellitus type 1 or 2, ischemic heart disease, or cancer did not influence the development of LCS (Table 2). Finally, we observed differences regarding the development of LCS between female and male COVID-19 patients. As such, obesity had stronger effect in women than in men and a pre-existing cancer diagnosis had a significant effect on the development of LCS in men but not women. In contrast, asthma and depression were significantly associated with LCS in female but not male COVID-19 patients (Table 2).

**Table 2:** Association between predefined variables and the incidence of long COVID syndrome in patients diagnosed with COVID-19 (multivariate logistic regression model)

Variable	Number of patients in the subgroup (*n*)	Proportion of patients with post-COVID-19 syndrome (*n*, %)	Total		Women		Men	
			Odds ratio (95% confidence interval)^1^	*p* value	Odds ratio (95% confidence interval)^1^	*p* value	Odds ratio (95% confidence interval)^1^	*p* value
Total	50,402	1708 (3.4)						
Age ≤ 30 years	10,443	213 (2.0)	Reference					
Age 31–45 years	12,963	379 (2.9)	1.33 (1.12–1.58)	0.001	1.29 (1.04–1.60)	0.023	1.39 (1.05–1.84)	0.021
Age 46–60 years	14,424	664 (4.6)	1.81 (1.54–2.13)	< 0.001	1.67 (1.36–2.05)	< 0.001	2.04 (1.56–2.67)	< 0.001
Age > 60 years	12,572	452 (3.6)	1.19 (0.99–1.43)	0.071	1.09 (0.86–1.39)	0.460	1.37 (1.01–1.86)	0.045
Female	27,512	1056 (3.8)	1.33 (1.20–1.47)	< 0.001	−		−	
Male	22,890	652 (2.9)	Reference		−		−	
Hypertension	12,898	634 (4.9)	1.31 (1.15–1.48)	< 0.001	1.27 (1.08–1.49)	0.004	1.39 (1.13–1.70)	0.001
Lipid metabolism disorder	8580	472 (5.5)	1.46 (1.28–1.65)	< 0.001	1.43 (1.21–1.68)	< 0.001	1.49 (1.22–1.81)	< 0.001
Diabetes mellitus type 1	364	15 (4.1)	1.00 (0.59–1.69)	0.987	0.98 (0.45–2.11)	0.950	0.99 (0.48–2.05)	0.978
Diabetes mellitus type 2	5060	233 (4.6)	0.93 (0.79–1.10)	0.389	0.80 (0.64–1.02)	0.069	1.10 (0.87–141)	0.440
Obesity	4995	271 (5.4)	1.25 (1.08–1.44)	0.003	1.28 (1.06–1.53)	0.010	1.19 (0.94–1.51)	0.153
BMI < 25.0 kg/m^2^	2521	136 (5.4)	Reference		Reference		Reference	
BMI 25.0–29.9 kg/m^2^	2693	138 (5.1)	0.95 (0.74–1.21)	0.662	0.92 (0.67–1.26)	0.611	1.14 (0.75–1.73)	0.543
BMI 30.0–34.9 kg/m^2^	1549	95 (6.1)	1.15 (0.88–1.50)	0.323	1.20 (0.85–1.69)	0.301	1.25 (0.79–2.00)	0.342
BMI ≥ 35.0 kg/m^2^	969	63 (6.5)	1.22 (0.90–1.66)	0.207	1.20 (0.83–1.73)	0.337	1.34 (0.76–2.36)	0.311
Ischemic heart diseases	3803	187 (4.9)	1.08 (0.91–1.29)	0.391	1.21 (0.96–1.52)	0.115	0.90 (0.69–1.18)	0.458
Asthma	4073	231 (5.7)	1.49 (1.28–1.73)	< 0.001	1.67 (1.39–2.00)	< 0.001	1.22 (0.93–1.59)	0.155
Chronic bronchitis/COPD	4399	187 (4.3)	0.93 (0.79–1.10)	0.379	0.82 (0.64–1.04)	0.056	1.13 (0.88–1.46)	0.328
Cancer	2605	132 (5.1)	1.21 (1.00–1.46)	0.054	1.04 (0.81–1.35)	0.737	1.46 (1.09–1.95)	0.012
Depression	8529	416 (4.9)	1.21 (1.07–1.37)	0.002	1.27 (1.09–1.47)	0.002	1.14 (0.92–1.41)	0.223

^1^The logistic regression analysis was adjusted for age, sex, comorbidities including hypertension, lipid metabolism disorder, diabetes mellitus, obesity, ischemic heart diseases, asthma, chronic bronchitis/COPD, cancer, depression. BMI values were available for 7732 patients

## Discussion

Our data suggest that lipid metabolism disorders and obesity but not diabetes represent strong age-independent risk factors for LCS. As the pathophysiology of LCS is presently unclear, this finding provides important information about a possible pathophysiological relationship of metabolic risks and the development and severity of LCS. This would support the hypothesis that obesity-related chronic inflammation and immune-metabolic processes promote not only severe clinical courses of acute SARS-CoV-2 infection [8], but also the development of LCS. In this context, it cannot be excluded that in our statistical analysis, there might have been an indirect association between severe courses of COVID-19 and the occurrence of LCS. However, it should be noted that the data source of outpatients with SARS-CoV-2 infection makes it unlikely that severe clinical courses had accumulated in our cohort of LCS patients. Moreover, diabetes or age > 60 years, known risk factors for severe courses of acute COVID-19 [9, 10], were not associated with LCS in our cohort, arguing against a linear concordance between risk-profiles of acute COVID-19 and LCS.

Post-acute sequelae (PAS) in the context of viral respiratory infections do not represent a fundamentally new observation, since PAS were already described as a consequence of other non-persistent viral infections in the pre-COVID era [11]. Of note, recent data suggest that the clinical symptoms, which are now referred to as LCS, likewise occur after infection with seasonal influenza [12]. Interestingly, metabolic factors are also discussed as potential risk factors for short and long-term mortality and morbidity for other viral infections as well [13–15], highlighting the general role of metabolic diseases as determinants for patients’ long-term outcome after viral infections. Besides metabolic risk factors, we identified other pre-existing medical conditions such as asthma, arterial hypertension and depression as important risk factors for the development of LCS. Our observation that female sex and patients’ age between 46 and 60 years indicate an increased risk of LCS is consistent with other published data from non-hospitalized [16] or hospitalized cohorts [17] of COVID-19 patients. Sigfrid et al. showed that women under age 50 were up to five times less likely to report feeling recovered and twice as likely to report worse fatigue than men of the same age [17]. A recent study of a cohort of healthcare workers (HCW) made observations that point in a similar direction to our data on a larger and more representative population, showing an OR of 1.6 for HCWs who were overweight and an OR of 3.7 for HCWs who had lung disease [18].

In contrast to previous studies of LCS, which have focused predominantly on specific patient groups and tended to study cohorts cared for at specialized COVID-19 centers, our study features a large cohort of COVID-19 outpatients that are representative for the sociodemographic situation in Germany and other high-income countries. However, we acknowledge some limitations. First, during the study period, LCS represented a novel diagnosis that evolved over time and had not yet been assigned to a specific ICD code. Clear diagnostic criteria as nowadays provided by the WHO were lacking, which may have led to overestimation or underestimation of LCS cases. Second, besides diagnosis of LCS in the original diagnosis text, we included diagnoses suggestive for LCS (e.g., “abnormalities of breathing”) that could also occur independently of COVID-19 and there is no valid information if these symptoms were associated with COVID-19 or not. In contrast, some diagnoses that are also consistent with LCS may not have been sufficiently accounted for. Moreover, data starting from March 2020 were used while LCS has really came to light at the end of 2020, which explains why this diagnosis was documented more often in the last months. Finally, we were unable to include a control group as the diagnosis LCS cannot occur in people who were not diagnosed with COVID-19 previously. Nevertheless, our database of currently more than 50,000 COVID-19 patients is a valuable source to identify risk factors for the development of LCS. The overlap with previously published results [16, 17] strengthens the validity of our results and supports the usability of our database in the context of LCS research.

In summary, since obesity and lipid disorders represent modifiable risk factors, our data suggest that lifestyle and metabolic interventions could be part of future strategies for pandemic preparedness. Moreover, our data clearly support the fact that patients with metabolic diseases should be considered as risk patients in all phases of COVID-19, and therefore, need a close clinical supervision even after overcoming the acute phase of COVID-19.
